# An audit of out of hours MRI scanning at a tertiary care referral hospital

**DOI:** 10.1186/1753-6561-9-S1-A53

**Published:** 2015-01-14

**Authors:** S Alnafisee, A O’Hare, J Thornton, P Brennan, S Looby

**Affiliations:** 1 Department of Neuroraudiology, Beaumont Hospital, Dublin 9, Ireland

## Introduction

The aim of this audit was to record the numbers of MRI scans performed out of hours or on-call in a busy tertiary care referral hospital. As this service continues to increase, we look at the trends and patterns in referrer pathways, the clinical indications for these studies, the types of studies performed, the results of these studies and the impact on patient management.

## Materials and methods

We retrospectively viewed the on-call MRI logbooks at Beaumont hospital and recorded all scans done this past decade. Regarding each study, the following major parameters were recorded; indication for study, requesting service, type of MRI performed, the result of each study and the impact on patient management.

## Results

A total of 1332 on-call MRI scans were performed on-call this past decade (2003-2012). The largest increase in scan numbers was from 2010-2011 and 2011-2012. The most frequent scan was that of the spine, followed by the brain. Fifty four percent of scans were positive, with a significant result altering patient management. The largest cohort of scans was referred by the neurosurgery service.

## Conclusion

There has been an exponential increase in out of hours MRI scanning over the past decade. This is an expensive service requiring several on-call staff. Despite the addition of a second MRI magnet at our hospital, this demand has continued to increase. For future planning of services, increasing MRI availability will be necessary, possibly in the form of an extended working day and/or in acquiring additional MRI magnets.

